# Supplementary figures and images for: A novel approach reveals that HLA class 1 single antigen bead-signatures provide a means of high-accuracy pre-transplant risk assessment of acute cellular rejection in renal transplantation
Source: BMC Immunol. 2019 Apr 27;20:11. doi: 10.1186/s12865-019-0291-2 (PMC6486998; doi:10.1186/s12865-019-0291-2)

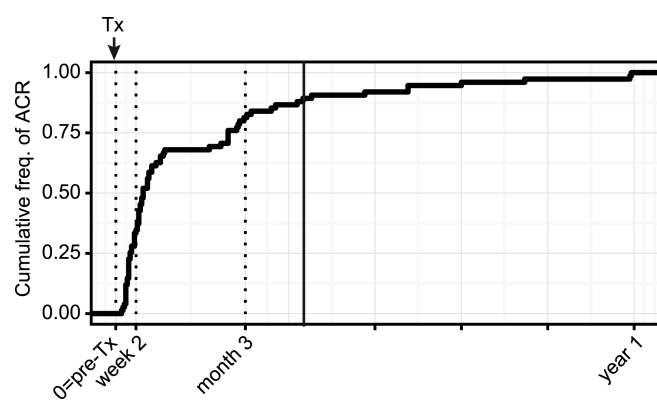

Supplement: Supplementary file 1 — Figure S1. Cumulative frequency of ACR through the first post-transplantation year. Frequency of patients in the ACR group who experienced at least one ACR event at different time points post-transplantation. At week 2 and month 3, 34% and 79% of patients in the ACR group had experienced an ACR event. (PDF 126 kb) [file 12865_2019_291_MOESM1_ESM.pdf]

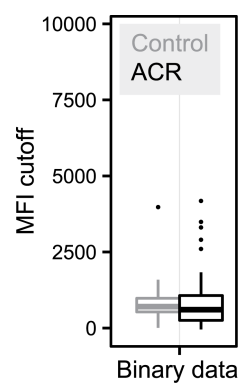

Supplement: Supplementary file 2 — Figure S2. Distribution of MFI cutoffs for generation of binary HLA class 1 SAB data. Illustrated are the MFI cutoff values of the 52 pre-transplant serum samples (control group = 18; ACR group, Rej = 34) of the binary HLA class 1 SAB data (1: presence of reactivity = above the MFI cutoff). (PDF 53 kb) [file 12865_2019_291_MOESM2_ESM.pdf]

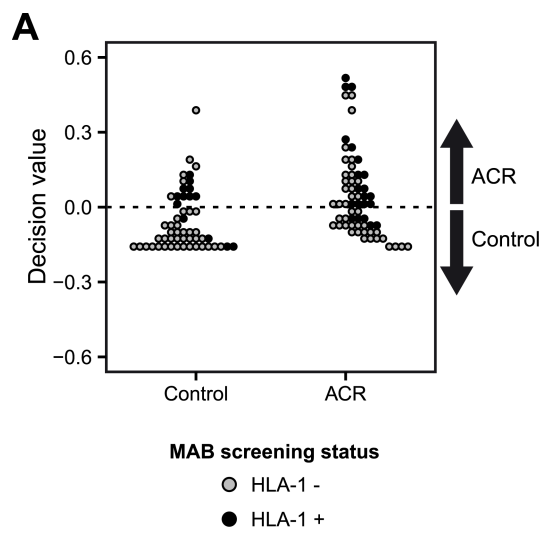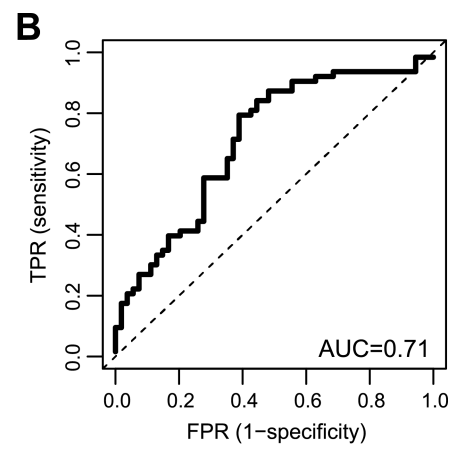

Supplement: Supplementary file 3 — Figure S3. Predictive performance of multiparameter ACR classification based on rank-normalized continuous pre-transplant MAB screening data. (A) Output of the classifiers decision function for each patient. The decision threshold is indicated by a dashed horizontal line. Patients with a decision value > 0 are classified as ACR, patients with a decision value < 0 are classified as control. Colors indicate whether patients tested positive (black) or negative (grey) for the presence of serum HLA-1 antibodies during MAB screening. (B) ROC curve of the multiparameter classifier. (PDF 211 kb) [file 12865_2019_291_MOESM3_ESM.pdf]
