# Supplementary material for: A novel approach reveals that HLA class 1 single antigen bead-signatures provide a means of high-accuracy pre-transplant risk assessment of acute cellular rejection in renal transplantation
Source: BMC Immunol. 2019 Apr 27;20:11. doi: 10.1186/s12865-019-0291-2 (PMC6486998; doi:10.1186/s12865-019-0291-2)
Supplement: Supplementary file 4 — Table S1. Baseline characteristics and medication details for the Harmony patient ACR-control sub-cohort. Baseline characteristics and medication details for the Harmony patient ACR-control sub-cohort.a (DOCX 18 kb) [file 12865_2019_291_MOESM4_ESM.docx]

Table S1. Study population characteristics and medication details of the Harmony cohort^a^

|  | | ACR | Control | p-value |
| --- | --- | --- | --- | --- |
| Number of kidney transplant recipients | | 77 | 80 | - |
| Age at time of transplantation (years) | | 54.9 ± 12.1 | 49.5 ± 12.6 | 0.005 |
| Body mass index at time of transplantation (kg/m^2^) | | 27.0 ± 5.3 | 25.6 ± 4.6 | 0.09 |
| Gender | Female | 31 (40.3%) | 29 (36.2%) | ns^b^ |
|  | Male | 46 (59.7%) | 51 (63.8%) |  |
| Type of donor | Living | 10 (13.0%) | 19 (23.8%) | 0.13^b^ |
|  | Deceased | 67 (87.0%) | 61 (76.2%) |  |
| Re-transplantation | | 5 (6.5%) | 1 (1.3%) | 0.11^c^ |
| HLA-A Mismatches | 0 | 25 (32.5%) | 33 (41.3%) | 0.18^b^ |
|  | 1 | 38 (49.4%) | 40 (50.0%) |  |
|  | 2 | 14 (18.2%) | 7 (8.7%) |  |
| HLA-B Mismatches | 0 | 13 (16.9%) | 24 (30.0%) | 0.09^b^ |
|  | 1 | 39 (50.6%) | 39 (48.7%) |  |
|  | 2 | 25 (32.5%) | 17 (21.3%) |  |
| HLA-DR Mismatches | 0 | 16 (20.8%) | 28 (35.0%) | 0.03^b^ |
|  | 1 | 39 (50.6%) | 41 (51.2%) |  |
|  | 2 | 22 (28.6%) | 11 (13.8%) |  |
| PRA = 0% |  | 70 (90.9%) | 75 (93.4%) | ns^c^ |
| Therapeutic Arm | A | 25 (32.5%) | 33 (41.3%) | ns^b^ |
|  | B | 29 (37.7%) | 21 (26.2%) |  |
|  | C | 23 (29.9%) | 26 (32.5%) |  |
| Cold ischemia time: only deceased donors (min) | | 704 ± 290 | 718 ±275 | 0.06 |

^a^Data are given as mean±standard deviation for quantitative variables and as number (frequency) for categorical variables. P values for quantitative variables were calculated by Mann-Whitney U test, for categorical variables either chi-squared (b) or Fisher’s exact test (c) were employed.

ACR: acute cellular rejection; ns: non-significant; PRA: panel reactive antibody.
