# Supplementary material for: A novel approach reveals that HLA class 1 single antigen bead-signatures provide a means of high-accuracy pre-transplant risk assessment of acute cellular rejection in renal transplantation
Source: BMC Immunol. 2019 Apr 27;20:11. doi: 10.1186/s12865-019-0291-2 (PMC6486998; doi:10.1186/s12865-019-0291-2)
Supplement: Supplementary file 5 — Table S2. Baseline characteristics and medication details for the patients with HLA MAB data.a (DOCX 20 kb) [file 12865_2019_291_MOESM5_ESM.docx]

Table S2. Study population characteristics and medication details of the patients with HLA MAB data^a^

|  | | ACR | Control | p-value |
| --- | --- | --- | --- | --- |
| Number of kidney transplant recipients | | 63 | 54 | - |
| Age at time of transplantation (years) | | 55.8 ± 11.3 | 49.7 ± 12.6 | 0.007 |
| Body mass index at time of transplantation (kg/m^2^) | | 26.8 ± 5.3 | 25.1 ± 4.4 | 0.09 |
| Gender | Female | 26 (41.3%) | 16 (29.6%) | ns^b^ |
|  | Male | 37 (58.7%) | 38 (70.4%) |  |
| Type of donor | Living | 8 (12.7%) | 12 (22.2%) | ns^b^ |
|  | Deceased | 55 (87.3%) | 42 (77.8%) |  |
| Re-transplantation | | 4 (6.3%) | 0 (0.0%) | 0.12^c^ |
| HLA-A Mismatches | 0 | 20 (31.7%) | 20 (37.0%) | ns^b^ |
|  | 1 | 30 (47.6%) | 29 (53.7%) |  |
|  | 2 | 13 (20.6%) | 5 (9.3%) |  |
| HLA-B Mismatches | 0 | 9 (14.3%) | 12 (22.2%) | ns^b^ |
|  | 1 | 34 (54.0%) | 29 (53.7%) |  |
|  | 2 | 20 (31.7%) | 13 (24.1%) |  |
| HLA-DR Mismatches | 0 | 10 (15.9%) | 20 (37.0%) | 0.009^b^ |
|  | 1 | 35 (55.6%) | 28 (51.9%) |  |
|  | 2 | 18 (28.6%) | 6 (11.1%) |  |
| PRA = 0% |  | 59 (93.7%) | 51 (94.4%) | ns^c^ |
| Therapeutic Arm | A | 22 (34.9%) | 20 (37.0%) | ns^b^ |
|  | B | 21 (33.3%) | 17 (31.5%) |  |
|  | C | 20 (31.7%) | 17 (31.5%) |  |
| Cold ischemia time: only deceased donors (min) | | 710 ± 292 | 729 ±293 | 0.10 |

^a^Data are given as mean±standard deviation for quantitative variables and as number (frequency) for categorical variables. P values for quantitative variables were calculated by Mann-Whitney U test, for categorical variables either chi-squared (b) or Fisher’s exact test (c) were employed.

ACR: acute cellular rejection; ns: non-significant; PRA: panel reactive antibody.
