# Supplementary material for: A novel approach reveals that HLA class 1 single antigen bead-signatures provide a means of high-accuracy pre-transplant risk assessment of acute cellular rejection in renal transplantation
Source: BMC Immunol. 2019 Apr 27;20:11. doi: 10.1186/s12865-019-0291-2 (PMC6486998; doi:10.1186/s12865-019-0291-2)
Supplement: Supplementary file 6 — Table S3. Single parameter pre-transplant prediction of ACR based on binarized HLA class 1 SAB data (fixed MFI threshold of 1000 MFI). (DOCX 18 kb) [file 12865_2019_291_MOESM6_ESM.docx]

**Table S3.** Single parameter pre-transplant prediction of ACR based on binarized HLA class 1 single antigen bead (SAB) data (fixed MFI threshold of 1000 MFI). *P-values are not corrected for multiple testing.

| Serotype | Fisher's exact test p-value* |
| --- | --- |
| A1 | 1.000 |
| A11 | 0.272 |
| A2 | 0.470 |
| A23 | 1.000 |
| A24 | 0.602 |
| A25 | 0.602 |
| A26 | 0.272 |
| A29 | 1.000 |
| A3 | 0.346 |
| A30 | 1.000 |
| A31 | 0.346 |
| A32 | 1.000 |
| A33 | 0.272 |
| A34 | 0.648 |
| A36 | 1.000 |
| A43 | 1.000 |
| A66 | 0.287 |
| A68 | 0.648 |
| A69 | 1.000 |
| A74 | NA |
| A80 | 0.425 |
| B13 | 0.682 |
| B18 | 0.285 |
| B27 | 0.543 |
| B35 | 1.000 |
| B37 | 1.000 |
| B38 | 0.543 |
| B39 | 1.000 |
| B41 | 1.000 |
| B42 | 1.000 |
| B44 | 1.000 |
| B45 | 1.000 |
| B46 | 0.602 |
| B47 | 1.000 |
| B48 | 0.150 |
| B49 | 0.543 |
| B50 | 0.538 |
| B51 | 0.399 |
| B52 | 0.081 |
| B53 | 1.000 |
| B54 | NA |
| B55 | 0.538 |
| B56 | 1.000 |
| B57 | 0.300 |
| B58 | 0.698 |
| B59 | 0.285 |
| B60 | 0.543 |
| B61 | 0.543 |
| B62 | 1.000 |
| B63 | 0.285 |
| B64 | NA |
| B65 | NA |
| B67 | 0.538 |
| B7 B | 0.648 |
| B71 | 1.000 |
| B72 | 0.543 |
| B73 | 1.000 |
| B75 | 0.648 |
| B76 | 0.327 |
| B77 | 0.543 |
| B78 | 1.000 |
| B8 B | 0.648 |
| B81 | 0.648 |
| B82 | 0.285 |
| Cw1 | 0.115 |
| Cw10 | 0.538 |
| Cw12 | 0.346 |
| Cw14 | 1.000 |
| Cw15 | 0.648 |
| Cw16 | NA |
| Cw17 | 0.139 |
| Cw18 | 1.000 |
| Cw2 | 0.114 |
| Cw4 | 1.000 |
| Cw5 | 1.000 |
| Cw6 | 1.000 |
| Cw7 | NA |
| Cw8 | NA |
| Cw9 | 0.538 |

ACR: acute cellular rejection; SAB: single antigen bead screening; NA: not applicable.
