# Supplementary material for: A novel approach reveals that HLA class 1 single antigen bead-signatures provide a means of high-accuracy pre-transplant risk assessment of acute cellular rejection in renal transplantation
Source: BMC Immunol. 2019 Apr 27;20:11. doi: 10.1186/s12865-019-0291-2 (PMC6486998; doi:10.1186/s12865-019-0291-2)
Supplement: Supplementary file 8 — Table S5. Summary of existing AR prediction models. (DOCX 19 kb) [file 12865_2019_291_MOESM8_ESM.docx]

| **Manuscript** | **Pubmed Identifier** | **Sensitivity [%]** | **Specificity [%]** | **Balanced Accuracy* [%]** | **Marker** | **Pretransplantation** | **Donor-independent** | **Cohort** |
| --- | --- | --- | --- | --- | --- | --- | --- | --- |
| Song et al. 2012 | 22389881 | 100 | 87 | 93.5 | HLA-2 DSA SAB | Yes | No | 5 ABMR (2 Mixed ABMR+ACR) vs. 22 No ABMR (4 ACR) |
| Hauser et al. 2005 | 15857922 | 93.3 | 89 | 91.15 | Urinary CXCL9 | No | - | 15 AR (14 ACR, 1 ABMR) vs. 54 No AR |
| Dong et al. 2006 | 16701175 | 87.1 | 91.8 | 89.45 | sCD30 | No | - | 49 AR vs. 182 No AR |
| Lefaucheur et al. 2010 | 20634297 | 90.6 | 85.4 | 88 | HLA DSA SAB | Yes | No | 32 ABMR vs. 370 No ABMR (18 ACR) |
| Simon et al. 2003 | 15239627 | 82 | 90 | 86 | Expression levels of Perforin and Granzyme B | No | - | 17 AR vs. 50 No AR |
| Shaikhina et al. 2017 | - | 81.8 | 88.9 | 85.35 | DSA HLA + IgG4 Levels + HLA Mismatches + Others | Yes | No | 46 ABMR vs. 34 No ABMR |
| **This work** | **-** | **76.5** | **88.9** | **82.7** | **Quantitative HLA-1 SAB** | **Yes** | **Yes** | **34 ACR vs. 18 No AR** |
| Riethmüller et al. 2010 | 20658760 | 75 | 90 | 82.5 | HLA-1 DSA SAB | Yes | No | 8 ABMR (Includes Chronic and Hyperacute; 5 Mixed) vs. 29 No ABMR (10 ACR) |
| Poggio et al. 2007 | 17460554 | 86 | 78 | 82 | Elispot Panel of Reactive Cells | Yes | Yes | 7 ACR (1 Mixed ABMR+ACR) vs. 23 No AR |
| Salvadé et al. 2016 | 27085791 | 70 | 93 | 81.5 | HLA DSA SAB | Yes | No | 10 ABMR vs. 14 No ABMR (2 ACR) |
| Mancebo et al. 2016 | 26773856 | 87.5 | 73.4 | 80.45 | Proportion of CD95+ CD8+ T Cells | Yes | Yes | 14 AR (2 ABMR) vs. 65 No AR |
| Pike et al. 2016 | 27148254 | 78 | 82.35** | 80.17 | PD1 Expression T cells | Yes | Yes | 9 AR (1 ABMR) vs. 17 No AR |
| Malheiro et al. 2015 | 25661873 | 85.7 | 73.1 | 79.4 | HLA DSA SAB | Yes | No | 14 ABMR (5 Mixed ABMR+ACR) vs. 26 No ABMR (2 ABMR) |
| Zhang et al. 2014 | 24935307 | 80 | 76.4 | 78.2 | Fractalkine and IP10 | No | - | 15 ACR vs. 35 No AR |
| Vondran et al. 2014 | 24931031 | 75 | 69.2 | 72.1 | sCD25, sCD30 and sCD44 | Yes | Yes | 7 ACR vs. 18 No AR (5 Borderline) |
| Nafar et al. 2009 | 19142743 | 70 | 71.7 | 70.85 | sCD30 | No | - | 23 AR vs. 180 No AR |

*Calculated by the authors of this study as the average of specificity and sensitivity.

** Not shown in the publication; calculated by the authors of this study.
